# What drives urban pandemic control performance in China? A configurational analysis of the resource-performance paradox

**DOI:** 10.3389/fpubh.2026.1849206

**Published:** 2026-06-15

**Authors:** Yexuan Zhou, Xianhong Huang, Jianing Wang, Huiyan Mao, Tao Sun

**Affiliations:** Department of Health Policy and Management, School of Public Administration, Hangzhou Normal University, Hangzhou, China

**Keywords:** bootstrap, digital governance capacity, fsQCA, TOE framework, urban pandemic control performance

## Abstract

**Background:**

The COVID-19 pandemic revealed a stark governance paradox in China: some resource-rich cities experienced governance failures, while relatively resource-scarce cities achieved effective control. This phenomenon challenges traditional linear resource determinism.

**Methods:**

To deconstruct this complexity, this study applies the Technology-Organization-Environment (TOE) framework and employs fuzzy-set qualitative comparative analysis (fsQCA), a configurational method capturing asymmetric causal relations, couple with Bootstrap analysis. Using data from 23 key Chinese cities from 2020 to 2022, we explore the conditions driving urban pandemic control performance.

**Results:**

The analysis identifies two distinct patterns leading to high performance. First, the “digital empowerment under resource constraints” pattern reveals that in resource-scarce contexts, digital governance capacity plays a core compensatory role, empowering prevention efforts despite material deficiencies. Second, the “holistic social synergy under resource abundance” pattern demonstrates that in resource-rich cities, effective resident wellbeing is the primary driver. Notably, digital governance assumes only a peripheral role in this configuration.

**Conclusion:**

These findings confirm that no single factor guarantees success; rather, performance depends on the alignment of governance strategies with local resource endowments. This study advises city managers to prioritize digital infrastructure in resource-scarce areas while focusing on social trust maintenance in affluent regions.

## 1 Introduction

In early 2020, the global outbreak of the COVID-19 pandemic posed a severe stress test to the public health emergency governance systems and governance capacities of countries worldwide (1). As of the end of August 2023, the World Health Organization reported over 770 million cumulative confirmed cases and more than 6.9 million deaths globally (2). In this unprecedented public health crisis, cities became the primary units of governance for pandemic prevention and control. Statistics indicate that approximately 90% of global confirmed cases were reported in urban areas, while this high proportion partly reflects the limited diagnostic and laboratory capacities in rural areas (leading to potential underreporting), the massive population scales and high connectivity significantly exacerbated the vulnerability of urban systems to pandemics (3). Consequently, in such a highly complex and uncertain environment, urban governance capacity has become a pivotal determinant of success in pandemic control and the rapid recovery of the socio-economy (4).

Yet, a retrospective review of China's anti-pandemic practices reveals a perplexing governance paradox: some mega-cities, endowed with robust economic foundations and stocks of premium medical resources, at times fell into operational paralysis and chaotic disorder (5) under the shock of the pandemic. Conversely, certain cities in the central and western regions, despite being relatively constrained in economic and medical resources, managed to achieve effective prevention and control (6). This counter-intuitive phenomenon directly challenges “resource determinism”, which assumes that a city's crisis response capacity is linearly and solely determined by its baseline material wealth and resource endowments. This observation necessitates a critical inquiry: under the multiple challenges of resource constraints, temporal and spatial limitations, and external pressures, exactly what specific configurations of key elements determine a city's operational resilience and prevention performance?

Despite the valuable insights provided by existing literature regarding how cities fulfill their pandemic prevention responsibilities, certain limitations remain, resulting in a fragmented understanding of effective control mechanisms. First, most studies are confined to exploring the “net effects” (7) of single factors, such as GDP or technology, on prevention performance. However, urban governance is a dynamic complex system where various elements often generate comprehensive effects through synergistic interactions (8). Traditional linear regression methods struggle to capture the causal complexity of multi-agent interactions (9), thereby overlooking the potential non-linear effects of multiple factors (10). Second, while prior studies pioneered configurational analyses of Chinese pandemic governance (6), they primarily focused on institutional elements, leaving the interactive role of resources and digital tools underexplored. Crucially, existing research neglects the contextual boundaries within which digital governance exerts its efficacy. Specifically, does digital technology function as a core condition driving performance across all contexts, or merely as a peripheral condition for achieving governance effectiveness in specific resource environments? The existing literature has failed to clarify this mechanism, leaving a research gap regarding how cities with different resource endowments achieve effective prevention through multi-factor synergy. This gap urgently calls for empirical exploration from a fresh perspective using novel methodological approaches.

Based on this, we construct an analytical model integrating the technology-organization-environment (TOE) framework (11). Although originally designed for technology adoption, the TOE framework is highly applicable to public health governance as it provides a holistic categorization to capture the synergy between technology, internal organizational mobilization, and external environmental pressures (12). Given the causal asymmetry, conjunctural causation, and complexity underlying pandemic control performance, this study employs fuzzy-set Qualitative Comparative Analysis (fsQCA) combined with bootstrap analysis to enhance the statistical robustness of the results. Rather than focusing on quantifying the impact of single variables, this study aims to identify the combinations of antecedent conditions that explain effective prevention. Specifically, using a sample of 23 key Chinese cities, this study seeks to answer two critical questions:

(1) Under the scenario of sudden public health emergencies, how do elements within the technology, organization, and environment dimensions achieve effective urban pandemic control through configurational synergy?
(2) Under different constraints of resource endowments and external pressures, what specific role (core or peripheral) does digital governance capacity play?

This study aims to provide specific theoretical insights and differentiated practical implications for building operational resilience in cities and responding to major public health events in the post-pandemic era.

## 2 Literature review

Existing literature regarding the drivers of pandemic control performance primarily centers on three key perspectives.

The first perspective focuses on the resource-based view. A consensus exists among most scholars that the level of economic development and the stock of medical health resources constitute the material foundation for withstanding shocks. For instance, complex economic structures can provide cities with stronger fiscal slack to resist the impact of the pandemic (13). Even though first-tier cities possess large populations, they hold sufficient resources to mitigate the adverse effects of the pandemic (14), thereby precluding cascading failures triggered by failures in specific urban structures or systems (15).

The second perspective highlights the technological governance view. With the advancement of the fourth industrial revolution, digital technology is regarded as a new engine for enhancing governance effectiveness. Big data and artificial intelligence have played a pivotal role in pandemic prevention across multiple countries, facilitating rapid diagnosis and risk forecasting of COVID-19(16). Some quantitative studies have also confirmed a negative correlation between the sophistication of smart infrastructure and the number of confirmed cases (17). Furthermore, Budd demonstrated the positive impact of digital technologies in contact tracing, interrupting transmission chains, and disseminating public health information (18), which substantially enhanced the efficiency and precision of public health responses. However, recent studies also warn that digital tools are not a panacea, noting that the “digital divide” can systematically exclude vulnerable groups (e.g., older adults) (19), while coordination barriers involving fragmented information systems often undermine data sharing and overall governance in disaster response (20).

The third perspective emphasizes the impact of social capital on prevention outcomes. Some scholars point out that social capital fosters community cohesion and trust (21), serving as a critical resource for residents in coping with crises (22). This view is further corroborated by Barrios (23), who found that residents and communities with stronger social capital are more inclined to adhere to social distancing measures, thereby decelerating the spread of the virus (24). Concurrently, local governments act as a key positive determinant of prevention performance. Governments possessing robust governance capacities can respond promptly and effectively, securing the supply of protective equipment and providing high-quality public health services to patients (25). Thus, in the post-pandemic Chinese context, social capital operates through interpersonal trust (26) and compliance to forge community resilience (27), and its protective effects are ultimately reflected in residents' subjective wellbeing as a tangible signature (28) of cooperative capacity.

## 3 Research framework

### 3.1 Urban pandemic control performance

Urban resilience refers to the capacity of an urban system to function normally under various shocks and stresses, thereby reducing vulnerability, adapting to environmental changes, and ensuring the continued operation of urban functions (29). Building on this concept, this study defines urban pandemic control performance as the outcome variable, which is aggregated from three-dimensional indicators: impact severity, lockdown status, and economic disturbance. Specifically, impact severity refers to the most severe single-wave outbreak experienced by the city during the observation period from January 1, 2020, to December 13, 2022 (noting that the National Health Commission of China ceased publishing data on asymptomatic cases starting from the 14th). Lockdown status indicates whether the city underwent prolonged (e.g., exceeding 1 week; specific rules are detailed in Supplementary Table 1), city-wide lockdowns, or “static management” during this period. Economic disturbance is measured by the deviation of the city's three-year average total retail sales of consumer goods. The calculation formula is as follows:


$$\begin{array}{l} D_{i}=\frac{\sum_{t=1}^{3}\left(R_{city,t}-R_{province,t}\right)}{3} \end{array}$$ (1)

where *D*_*i*_ represents the three-year average deviation for city *i*;*R*_*city, t*_ denotes the annual growth rate of total retail sales of consumer goods for the city in year *t*; *R*_*province, t*_ denotes the annual growth rate of total retail sales of consumer goods for the province where the city is located in year *t*.

### 3.2 TOE framework

**Figure 1:**
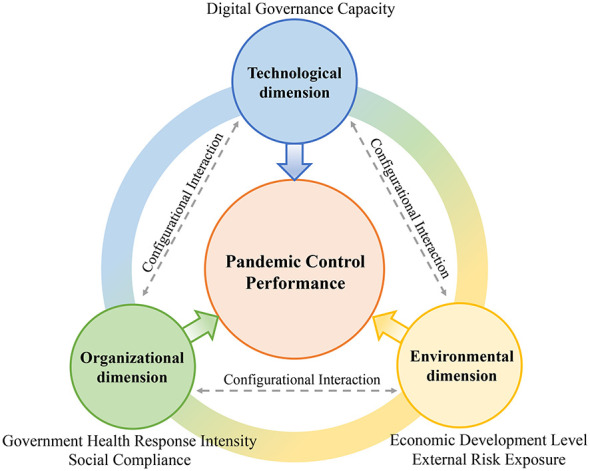
Theoretical model framework diagram.

Pandemic control is a complex systemic undertaking involving multi-agent participation, multi-resource allocation, and multi-level coordination. Consequently, theoretical perspectives focusing on a single dimension offer limited explanatory power. To address this, this study introduces the Technology-Organization-Environment (TOE) framework. This framework, which integrates insights from innovation diffusion theory and the technology acceptance model (30), emphasizes the interaction among technological, organizational, and environmental dimensions, making it highly effective for explaining the complex causalities of intricate social phenomena (31). Therefore, based on these three dimensions, we selected five key condition variables to explore their configurational effects on pandemic control performance: digital governance capacity (Technology), government health response intensity and social compliance (Organization), economic development level and external risk exposure (Environment). The theoretical model framework of this study is illustrated in Figure 1.

#### 3.2.1 Technological dimension

*Digital governance capacity*. In the digital era, technological empowerment has emerged as a core driver for enhancing urban resilience (32). This variable is designed to reflect the comprehensive level of each city in utilizing technological means such as big data, cloud computing, and artificial intelligence for pandemic control during the period from 2020 to 2022. By leveraging digital tools such as contact tracing, pandemic maps, and city brain systems, governments can effectively overcome information asymmetry in crisis response, thereby achieving precise identification of at-risk populations and precise allocation of medical resources. Given the absence of unified official quantitative indicators, this study employed systematic content analysis on special pandemic reports sourced from authoritative media, including the official government websites of each city (or province), People's Daily Online, and Xinhua Net.

#### 3.2.2 Organizational dimension

##### 3.2.2.1 Government health response intensity

As the organizational entity of governance, the city system relies heavily on the resource mobilization capacity of the government to determine the effectiveness of prevention and control during sudden public health crises (33). Health response intensity reflects the local government's willingness for emergency disposal and the agility of the health system under crisis scenarios. During the outbreak of a pandemic, it measures whether the government can rapidly adjust the fiscal budget structure and significantly increase health expenditures to control the speed and scope of transmission and maintain the normal operation of social order.

This study measures the government's response intensity using the growth rate of the proportion of fiscal health expenditure. The specific calculation method is as follows: calculate the average proportion of health expenditures in the city's total general public budget expenditures over the 3 years (2020-2022), and subtract the proportion from the pre-pandemic year (2019). This difference reflects the degree of fiscal resource inclination and the magnitude of the government's response under the shock of the pandemic.

##### 3.2.2.2 Social compliance

Within the organizational dimension of the TOE framework, social compliance serves not merely as an indicator of people's livelihood but as a comprehensive embodiment of urban social resilience. Drawing on legitimacy theory, interactions between the government and the public may be viewed as a process of legitimacy acquisition and accumulation. Organizations possessing high legitimacy are better positioned to garner active public support to withstand environmental shocks (34). When facing pandemic challenges, this widespread social recognition translates into active compliance behavior, drastically reducing resistance to policy implementation and thereby significantly lowering governance costs.

To operationalize this construct, we use resident wellbeing as a proxy variable for social compliance. This study adopts the “Effective Urban Resident Wellbeing Index System based on Big Data” launched by the China happy cities lab for measurement. This index is not simply a measure of subjective feelings but a comprehensive evaluation system covering nine primary indicators, including education, healthcare, residents' quality of life, transportation, and safety, along with hundreds of sub-dimensions. Inclusion in the list of “China's Happiest Cities” signifies that the city maintains a high level of public service supply quality and social trust (a core element of social capital), reflecting its comprehensive advantages in urban governance and public wellbeing. Therefore, high wellbeing essentially represents a high-quality foundation for social governance, which ultimately fosters the high social compliance required during pandemic crises.

#### 3.2.3 Environmental dimension

##### 3.2.3.1 Economic development level

The level of economic development constitutes the material foundation for cities to cope with sudden public health crises. During the pandemic, this economic advantage not only supports high investment in medical supplies and the construction of comprehensive medical infrastructure (35) but also buffers the economic shock to residents' livelihoods caused by work stoppages during lockdowns, thereby providing solid support for the ground-level implementation of prevention and control measures (36). Based on this, this study selects the 3-year average per capita GDP of the permanent resident population as the indicator for measuring the city's economic development level. Compared to total economic volume, the per capita indicator eliminates the interference of population scale, more accurately reflecting the actual level of economic security enjoyed by each citizen. Data were derived from the Statistical Yearbook of each city.

##### 3.2.3.2 External risk exposure

External risk exposure reflects the pressure of imported cases faced by the city. While modern transportation networks enhance the efficiency of resident mobility, they objectively provide “express lanes” for the rapid diffusion of the virus (37). As regional or even international hubs, cities face large-scale and highly complex population flows, which significantly increase the risk of trans-regional viral input. Given the dominant role of air passenger transport in long-distance virus transmission, this study selects civil aviation passenger throughput to measure the level of external risk exposure. The 3-year average airport passenger throughput is used as an indicator of the city's normalized mobility pressure. Data were derived from the Statistical Bulletin of Civil Aviation Airport Production issued by the Civil Aviation Administration of China.

In summary, the five condition variables mentioned above do not operate in isolation during pandemic prevention and control; rather, they are intertwined and interlocking. For instance, a high-risk environment (indicated by high airport passenger throughput) may require stronger digital technology to counterbalance the pressure, whereas regions with a weak economic foundation may rely more heavily on social compliance to compensate for resource deficits. Therefore, it is necessary to introduce the configurational analysis method to systematically deconstruct how multiple factors act synergistically to achieve effective control performance.

## 4 Research design

### 4.1 Fuzzy-set qualitative comparative analysis

Given that the effectiveness of urban pandemic control is the result of the synergistic interplay of multiple factors, characterized by typical “multiple conjunctural causation” and “causal asymmetry”, traditional linear regression methods struggle to effectively uncover the underlying internal mechanisms. Therefore, this study employs the fuzzy-set qualitative comparative analysis (fsQCA) method. By calibrating the data to retain the variation in degree of continuous variables, this approach facilitates configurational comparisons to reveal the mechanisms of multiple conjunctural causation. All analyses in this study were conducted using the QCA package in RStudio.

### 4.2 Data sources

This study selected 23 prefecture-level cities (including 15 provincial capitals) that are representative in terms of pandemic control practices as the research sample. To minimize selection bias, the inclusion criteria were strictly defined based on three dimensions: (1) Administrative and economic significance, ensuring the sample covers major urban hubs that faced severe pandemic stress; (2) Geographical representativeness, ensuring a balanced distribution across different economic zones to reflect variations in resource endowments; and (3) Data availability, ensuring complete and continuous statistical records across all corresponding databases for the 2020–2022 period. Geographically, the sample consists of 11 cities from the eastern region, 4 from the central region, 3 from the northeast region, and 5 from the western region, covering a total of 18 provincial-level administrative regions in China. Data for the condition variables were derived from the Statistical Yearbook of each city, the Statistical Bulletin of Civil Aviation Airport Production issued by the Civil Aviation Administration of China (CAAC), and the “China's happiest cities” list. Data constituting the outcome variable were sourced from provincial and municipal statistical yearbooks, the official websites of health commissions, and briefings from authoritative news media. All the aforementioned data sources are authoritative and publicly accessible online. See Table 1 for details.

**Table 1:** Variable definitions and data sources.

Dimension	Variable name	Measurement indicator	Data source
Outcome variable	Pandemic control performance	Aggregated by impact severity, lockdown status, and economic disturbance	Provincial and municipal statistical yearbooks, health commissions, authoritative news media, etc.
Technology	Digital governance capacity	Digital governance capacity	Official government websites, *People's Daily Online, Xinhua Net*
Organization	Government health response intensity	Growth rate of the proportion of fiscal health expenditure	Municipal statistical yearbooks
	Social compliance	Effective urban resident wellbeing	“China's happiest cities” list
Environment	Economic development level	Per capita GDP	Municipal statistical yearbooks
	External risk exposure	Airport passenger throughput	*Statistical Bulletin of Civil Aviation Airport Production*

### 4.3 Variable calibration

In this study, the thresholds for the fuzzy-set calibration of both outcome and condition variables are set at 0.05 (full non-membership), 0.50 (crossover point), and 0.95 (full membership).

#### 4.3.1 Outcome variable

First, we performed fuzzy-set calibration on the three sub-dimensions: impact severity, lockdown status, and economic disturbance. These were inversely transformed to measure “high-level pandemic control performance”. Specifically, we calculated: Low_Impact_fs=1-Impact_fs (representing low pandemic impact severity); No_Lockdown_fs=1-Lockdown_fs (representing the absence of large-scale, prolonged lockdowns); Low_Disruption_fs=1-Disruption_fs (representing low socio-economic disturbance). These were then aggregated into the final urban pandemic control performance (Performance_fs).

Regarding the weighting logic, priority was assigned as follows: “No_Lockdown_fs” was assigned the highest weight (45%), as prolonged lockdowns exert the most profound negative impact on socio-economics (38), social order (39), and individual psychology (40). “Low_Impact_fs” was assigned the second-highest weight (35%), as it directly relates to human health and life (41), and the bearing capacity of the medical system (42). “Low_Disruption_fs” was assigned the remaining weight (20%), reflecting urban economic resilience and recovery capabilities (43).

Based on the aforementioned priority settings, the aggregation rules are as follows: if the calibrated values of all three sub-dimensions are not higher than 0.3, the case is judged as “extremely low performance” and assigned a value of 0.05. If all values are not lower than 0.7, the case is judged as “extremely high performance” and assigned a value of 0.95. For the remaining cities, the value is calculated using the weighted average method. The formula is:


$$\begin{array}{l} \;Performance\text{\_}fs=\left(0.35\times Low\text{\_}Impact\text{\_}fs\right)+\\ \left(0.45\times No\text{\_}Lockdown\text{\_}fs\right)+\left(0.20\times Low\text{\_}Disruption\text{\_}fs\right). \end{array}$$ (2)

Finally, this study anchored the crossover point of Performance_fs at 0.50. That is, a membership scores greater than 0.50 is defined as “high-level prevention and control performance”, while a score below 0.50 represents “low-level performance”. Specific anchor point calibration and setting rationales are detailed in Supplementary Table 1.

#### 4.3.2 Condition variables

This study selected a total of five condition variables to analyze their impact on urban pandemic control performance from five key dimensions: economic development level, government health response intensity, external risk exposure, social compliance and digital governance capacity. For the first three variables, raw data were derived from the averages between 2020 and 2022 (government health response intensity was calculated as the average change relative to the 2019 baseline). These variables employ fuzzy-set calibration, where a higher score indicates a higher degree of presence of the condition. The latter two variables employ crisp-set calibration: EURW_cs (Effective Urban Resident Wellbeing): assigned a value of 1 if the city was selected in the “China's happiest cities” list for at least 2 years; otherwise, assigned 0. DigitalGov_cs (digital governance capacity): assigned a value of 1 if a local government-led digital governance platform or health code system played a pivotal role during the pandemic; otherwise, assigned 0.

The setting of anchor points in this study comprehensively considers macro data published by authoritative institutions such as the national bureau of statistics, relevant theoretical consensus, and the actual data distribution characteristics of the cases. Table 2 summarizes the anchor point settings for each condition variable. Specific anchor points and the rationale for their setting are detailed in Supplementary Table 2.

**Table 2:** Summary of calibration anchor points for condition variables.

Condition variable (abbreviation)	Dimension	Full non-membership	Crossover point	Full membership
GDPpc (CNY 10,000)	Economic development level	8	12.5	16.5
HealthExpChange	Government health response intensity	0.007	0.012	0.04
Passenger throughput (10,000 passengers)	External risk exposure	200	1,000	4,000
EURW (0/1)	Social compliance			
DigitalGov (0/1)	Digital governance capacity			

EURW and DigitalGov are calibrated as crisp sets (0 or 1), thus they do not have intermediate fuzzy-set anchors.

### 4.4 Robustness check strategy: bootstrap analysis

The robustness of QCA results is known to be highly sensitive to case composition. Particularly in small-N studies, the exclusion of individual cases or minor adjustments to parameters may lead to drastic fluctuations in the results. To enhance the credibility of the findings, this study constructs a multi-layered robustness check procedure. In addition to the conventional consistency threshold perturbation test and a sensitivity analysis of the outcome variable's weighting scheme, we further introduce the bootstrap resampling statistical framework. Specifically, we first evaluate whether the identified configurations are sensitive to the subjective weights assigned to the outcome variable's sub-dimensions. Then, we perform 1,000 resamplings on the original dataset to observe the frequency of occurrence of core configurations within the random samples, thereby identifying robust causal configurations.

## 5 Results

### 5.1 Analysis of necessary conditions

First, this study conducted an analysis of necessary conditions for the five antecedent conditions using the R environment. According to Fiss (44), a condition can be deemed necessary when its consistency score exceeds 0.9. As shown in Table 3, all consistency scores are below 0.9. This indicates that no single factor alone constitutes a necessary condition for achieving high pandemic control performance, which perfectly aligns with the core premise of configurational “equifinality”(45). This finding further corroborates that pandemic control is a complex systemic undertaking, necessitating the examination of concurrent synergistic effects among multiple factors.

**Table 3:** Analysis of necessary conditions.

Condition	Consistency	Coverage
~GDPpc_fs	0.542	0.660
GDPpc_fs	0.639	0.866
~HealthExpChange_fs	0.651	0.878
HealthExpChange_fs	0.664	0.732
~DigitalGov_cs	0.207	0.790
DigitalGov_cs	0.793	0.561
~EURW_cs	0.497	0.549
EURW_cs	0.503	0.857
~Passengerthroughput_fs	0.708	0.846
Passengerthroughput_fs	0.622	0.779

### 5.2 Sufficiency analysis

This study employed RStudio to construct the truth table. Given the limited sample size (small-N), the frequency threshold was set to 1, meaning that configurations covering at least one case were included in the analysis. The raw consistency threshold was set to 0.8. Furthermore, to prevent simultaneous subset relations, which a configuration might be a subset of both the outcome and its absence, the PRI (Proportional Reduction in Inconsistency) consistency threshold was set to 0.70 (46). Any configuration with a PRI score below this benchmark, even if possessing high raw consistency, was excluded from the logical minimization analysis. The detailed truth table is presented in Supplementary Table 3.

In the configurational analysis, this study reports the intermediate solution. This solution is chosen because it incorporates logical remainders consistent with theoretical expectations while avoiding the risk of over-simplification (47). The directional expectations were set as follows: the presence of GDP per capita, digital governance capacity, and effective urban resident wellbeing is expected to contribute to high prevention and control performance. Conversely, passenger throughput and growth rate of health expenditure were set to their absence to reflect the control of imported risks and emergency costs. The rationale for anticipating a low level (denoted by ~) of health expenditure growth is that effective prevention relies more on existing infrastructure reserves and precision governance during non-crisis periods, rather than on reactive, surge-style fiscal remediation following an outbreak. An excessively high growth rate may, in fact, imply a passive response triggered by a failure in initial containment.

As shown in Table 4, the overall solution consistency is 0.919, the overall solution coverage is 0.520, and the overall PRI is 0.869, indicating that the model possesses robust explanatory power for pandemic control performance. The results reveal a total of three prevention pathways. Based on the core logic of the configurations, this study categorizes them into two distinct patterns, named as follows:

**Table 4:** Configurations for high-level pandemic control performance.

Conditions	Pattern 1: digital empowerment	Pattern 2: holistic synergy	
	C1	C1	C2
GDP pc	×	•	•
Passenger throughput	⊗		°
HealthExp Change	⊗	°	
Effective UR wellbeing	×	•	•
Digital Gov	•	°	°
Consistency	0.951	0.922	0.878
Raw coverage	0.161	0.292	0.268
Unique coverage	0.161	0.091	0.066
Overall consistency	0.919		
Overall coverage	0.520		

This table presents the intermediate solution of the configurational analysis. “•”indicates the presence of a core condition; “⊗”indicates the absence of a core condition; “°”indicates the presence of a peripheral condition; “ × ”indicates the absence of a peripheral condition. Blank spaces indicate a “don't care” situation.GDP pc, Economic Development Level; Passenger throughput, External Risk Exposure; HealthExp Change, Government Health Response Intensity; Effective UR Wellbeing, Social Compliance; Digital Gov, Digital Governance Capacity.

Pattern 1: digital empowerment under resource constraints. This pattern (consistency = 0.951, coverage = 0.161) primarily occurs in cities characterized by weak economic foundations, limited government health fiscal investment, and average effective urban resident wellbeing. In an environment of “dual resource scarcity”, cities rely on robust digital control measures as the core driver, combined with low external risk input, to compensate for resource deficits.

Pattern 2: holistic social synergy under resource abundance. This pattern comprises two sub-paths that exhibit high similarities, both featuring high GDP per capita, high digital governance capacity and high effective urban resident wellbeing. Path 1 (consistency = 0.922, coverage = 0.292) shows that, building on the aforementioned foundation, the government achieves effective resource mobilization and collaborative governance through relatively high health fiscal investment.

Path 2 (consistency = 0.878, coverage = 0.268) indicates that even under extremely high external risk input, cities can still achieve excellent prevention performance by relying on a robust economic foundation, advanced digital technology and effective social synergy. This pattern explains or reflects the comprehensive governance advantage of developed cities, where technology, organization, and environment form a complementary support system.

### 5.3 Robustness check

Common methods for QCA robustness analysis include adjusting calibration thresholds, changing case frequency thresholds, and varying consistency thresholds (48). Given the limitation of the sample size (*N* = 23) in this study, and to avoid the methodological complexity associated with time-consuming and unstable iterative threshold calibration sensitivity analysis, we focused on truth table threshold sensitivity analysis and bootstrap robustness analysis. Based on the original thresholds (consistency = 0.80, PRI = 0.70), we tested combinations that were both looser (0.75/0.65) and stricter (0.85/0.75). The results showed that the path structure, core conditions, and parameter metrics of the configurations did not undergo substantial changes (see Table 5). This demonstrates that the research results possess extremely high robustness and are unaffected by the subjective selection of thresholds.

**Table 5:** Comparison of robustness check results.

Test criteria	Threshold settings	No. of paths	Structure change	Overall Cons.	Overall cov.	Conclusion
Original	Cons. 0.80/PRI 0.70	3	Baseline	0.919	0.520	—
Strict	Cons. 0.85/PRI 0.75	3	Consistent	0.919	0.520	Robust
Loose	Cons. 0.75/PRI 0.65	3	Consistent	0.919	0.520	Robust

Furthermore, to eliminate potential biases arising from the subjective weighting in the construction of the composite outcome variable, we conducted an additional sensitivity analysis by applying an equal weighting scheme (assigning 33.3% to each of the three sub-dimensions). As detailed in Supplementary Table 4, the re-run of the fsQCA procedure under the alternative equal weighting scheme yielded the identical three configurational pathways. The overall consistency (0.927) and coverage (0.526) remained highly stable and even slightly improved. This empirical evidence firmly demonstrates that our configurations represent robust empirical patterns rather than artifacts of subjective weighting selections.

To further overcome potential random biases inherent in small-N samples, this study utilized RStudio to conduct a bootstrap test, performing 1,000 random resamplings with replacement on the original sample. The results indicate that the intermediate solution ranked first among all potential solutions with a frequency of 16.4%. The sample mean consistency was 0.959 with a standard deviation (SD) of only 0.037; the mean coverage was 0.523 with an SD of 0.063. This indicates that the configurational structure is not an accidental result. Even when the sample undergoes random perturbations, the consistency and explanatory power of the model remain at an extremely high and stable level with minimal fluctuation, suggesting that the conclusions of this study possess statistical robustness.

## 6 Discussion

The empirical results from the fsQCA reveal that effective urban pandemic control is not the product of isolated linear factors, but rather emerges from complex configurational synergies across the technology, organization, and environment dimensions. Building upon the three sufficient pathways identified in the results, this section further unpacks the theoretical mechanisms underlying these overarching patterns and discusses their broader implications.

Drawing on the technology-organization-environment (TOE) framework, this study selects five condition variables to examine the configurational conditions facilitating effective urban pandemic control. By applying fuzzy-set qualitative comparative analysis, this study explores the complex causal mechanisms underlying the interplay of multiple conditions in determining prevention and control performance. The empirical results indicate that no single condition alone can account for high-level pandemic control performance. Instead, achieving effective prevention exhibits the characteristic of equifinality (45). Through the analysis of necessary conditions and sufficient configurations, this study identifies two distinct patterns, characterized as “digital empowerment under resource constraints” and “holistic social synergy under resource abundance”.

The first pattern reveals how digital governance plays a core role in resource-constrained contexts: it effectively compensates for the deficiency of other resources, thereby empowering prevention efforts and enhancing performance. In this pathway, digital governance capacity appears as a core condition, whereas the growth of government health expenditure presents as an absence. It is crucial to adopt a conditional interpretation here to avoid conflating two different scenarios: a wealthy city with a high health-spending baseline and low growth, vs. a resource-constrained city with a low baseline and low growth. We argue that this “low growth in health expenditure” should not be simply interpreted as insufficient investment; rather, it possesses dual implications:

On the one hand, it reflects the objective resource constraints faced by cities with moderate economic development. Being at a disadvantage in terms of fiscal capacity and resource stock (49, 50), these cities struggle to replicate the resource-intensive configuration model of economically developed regions. Therefore, their “low growth” is not an artifact of a massive pre-existing resource baseline, but rather dictates an urgent need to leverage digital technology to bridge the resource gap. In this context, a city's digital capacity is not a mere auxiliary enhancement, but has become the core pillar for crisis response.

On the other hand, this also manifests the effectiveness of early-stage pandemic prevention. Unlike traditional strategies that rely heavily on massive post-disaster remedial investments, these cities proactively leverage preemptive digital technologies to sever transmission chains at the nascent stage of the crisis. With moderate economic strength and resource foundations, these cities strategically prioritize “prevention over cure”, thereby effectively avoiding the exorbitant remedial costs associated with later-stage risk escalation.

Therefore, the “low input” in this pattern does not signify governance failure but represents a form of cost-effective governance enabled by digital empowerment. A typical case is Hefei, China. According to our QCA results, this city exemplifies a configuration where technology-led strategies compensate for resource constraints. Over the past decade, the city has experienced rapid technological development, though its economic strength still has significant room for improvement. During the pandemic, it fully utilized technological means to support material resources, forming an effective and adaptive prevention strategy that achieved positive results.

The second pattern indicates that when cities possess abundant fiscal and medical resources, effective urban resident wellbeing becomes the key determinant for achieving high performance. Interestingly, within this configuration, digital governance capacity transitions into a peripheral condition. This implies that in such contexts, resource abundance expands the spectrum of governance tool choices available to the city (51). Consequently, digital technology functions more as an auxiliary instrument for optimizing resource allocation rather than as a decisive core force. Drawing upon our configurational findings, effective urban resident wellbeing reflects high levels of social trust and public compliance. It forms a complementary relationship with material resources, mirroring the proactive participation of the populace in pandemic prevention within the urban field. While abundant material supplies provide a “hard” guarantee, favorable social compliance significantly enhances policy implementation efficiency and reduces governance friction (52). Together, these factors bolster the city's system resilience in coping with sustained external shocks. As empirical illustrations of this specific configurational pattern, cities such as Hangzhou, Wuxi, and Changsha provide real-world insights into how this combination of resources and social compliance operates in practice.

It is crucial to distinguish the nuanced mechanisms between path 1 and path 2 within this “holistic social synergy” pattern. While both share the foundational conditions of high economic development and social compliance (proxied by effective urban resident wellbeing, EURW), path 1 utilizes relatively high health fiscal investments to rapidly convert material strength into governance outcomes. In contrast, path 2 illustrates how cities can withstand extremely high external risk exposure through the synergistic interplay of economic foundations, digital tools, and social compliance, even without exceptional surges in health expenditures. This nuance highlights that resource-abundant cities can strategically adapt their governance focus: either relying on rapid internal resource mobilization or leveraging comprehensive systemic resilience to buffer external risks, depending on their specific exposure levels.

Cities exemplifying this overarching pattern, such as Hangzhou and Wuxi, vividly illustrate this synergy in practice. For instance, according to municipal statistical communiqués (53, 54), both cities maintained exceptionally high per capita GDP and recorded substantial added-value in their digital economy core industries during 2020-2022, providing robust fiscal and technological slack. Concurrently, their long-term presence on the “China's Happiest Cities” list (55) reflects a high baseline of institutional trust, enabling them to execute public health mandates with minimal administrative friction.

### 6.1 Theoretical contributions

The theoretical contributions of this study are primarily reflected in the following three aspects: First, by introducing the TOE framework and a configurational perspective into the governance of sudden public health crises, this study transcends the limitations of previous research that focused on the “net effects” of single variables. Prior literature predominantly employed regression analysis to explore the impact of isolated factors such as economy (56) or policy (57, 58), largely overlooking the complex interactions among these elements (45). This study reveals that under extreme pressure, technological, organizational and environmental elements do not function through simple addition; rather, they achieve functional complementarity through specific combinations. This perspective elucidates why cities with differing resource endowments generate vastly different prevention strategies and outcomes, even when facing identical external pressure scenarios and uniform central prevention policies.

Second, we clarify the dual roles of digital governance in crisis response. While existing studies generally acknowledge the importance of digital tools (59–61), few discuss the boundary conditions of their effectiveness. In contrast, this study finds that the role of digital technology is highly contingent upon the resource environment in which it is embedded, it does not assume the same role or function across all contexts. In configurations characterized by a scarcity of material and economic resources, digital technology manifests as a core enabling force that fills gaps, allowing for more flexible prevention strategies and maximizing strengths while avoiding weaknesses. Conversely, in resource-abundant configurations, it transforms into an auxiliary factor that reinforces existing advantages. This finding refines theoretical discussions on technological empowerment, demonstrating that the release of digital technology's efficacy requires alignment with organizational resource endowments, rather than serving as an absolute core driver in every scenario.

Third, integrating urban resident wellbeing into the analytical framework expands the explanatory dimensions of crisis governance. Traditional emergency management research often focuses on perspectives such as resources (62), policies (63), or crisis life cycles (64). However, this study identifies that in resource-abundant pathways, effective urban resident wellbeing is the key to achieving high performance. High wellbeing is often accompanied by stronger social trust among residents. In times of crisis, this latent government reputation can potentially translate into public compliance behavior (65, 66) and public service motivation (67), thereby reducing emergency response costs and social friction.

### 6.2 Policy implications

Based on the aforementioned findings, this study offers the following practical implications for city managers:

First, avoid blind imitation and formulate differentiated strategies tailored to local resource endowments. For cities in the central, western, and northeastern regions with moderate economic development, given that their fiscal and medical resources are objectively limited, they should not forcibly replicate the high-input, high-consumption prevention models of developed regions. Instead, they should channel limited budgets toward digital infrastructure. By leveraging the predictive and scheduling capabilities of big data to empower prevention efforts, digitalization can become the key to compensating for shortages in manpower and materials, thereby achieving low-cost, precision prevention.

Second, value the accumulation of urban reputation capital and build human-centric, resilient cities. For developed cities in the eastern region, the mere accumulation of resources has encountered diminishing marginal utility (68, 69). City managers must realize that residents' sense of gain and wellbeing are not merely the desired outcomes of urban operation but also intangible assets for sustainable development and a latent force for crisis response. Social trust, accumulated through improved public services during routine governance, can be activated once an emergency state is declared, subsequently transforming into powerful social mobilization capacity.

Finally, adopt a dynamic and contingent governance perspective (70, 71). City managers should flexibly adjust governance strategies according to their own resource constraints and changes in external risks. They should leverage technology to overcome difficulties when resources are scarce, while prioritizing social synergy when resources are abundant, striving to find the optimal balance of governance efficacy through dynamic adjustment.

## 7 Conclusion and limitation

### 7.1 Conclusion

Drawing on the TOE framework, this study employed the fsQCA method combined with Bootstrap robustness checks to conduct a configurational analysis of pandemic control performance in 23 key Chinese cities. The main conclusions are as follows:

First, addressing the first research question regarding how elements across the technology, organization, and environment dimensions achieve effective urban pandemic control through configurational synergy, this study finds that the achievement of high-level pandemic control performance exhibits the typical characteristic of “equifinality”. For a country with vast internal differences in resource conditions, prevention strategies should be tailored to local contexts. The study confirms that no single technological, organizational, or environmental element constitutes a necessary condition for effective prevention. Cities with differing resource endowments can achieve equivalent goals through differentiated combinations of elements. Specifically, two typical pathways exist: first, the “digital empowerment” pattern, which relies on digitalization to compensate for material deficits under resource constraints; second, the “holistic social synergy” pattern, which relies on economic foundations and social compliance under resource abundance.

Second, regarding the specific role of digital governance capacity under different resource constraints, this study demonstrates that it exhibits differentiated positioning across different resource contexts. Through configurational comparison, this study finds that the efficacy of digital technology depends on the city's own resource structure. In cities with scarce medical and fiscal resources, technology plays a core role in maintaining the operation of the prevention system at a low cost. Conversely, in resource-abundant cities, technology plays more of a peripheral role, acting as a tool to optimize resource allocation.

Third, preemptive technology governance provides a viable pathway for regions with moderate economic levels to break free from resource dependence. Empirical results indicate that by establishing agile digital warning and precise control systems, cities can effectively sever the pandemic transmission chain without relying on massive fiscal inputs. This conclusion not only provides empirical evidence for the governance of sudden public health emergencies but also offers important references for how these regions can achieve agile governance under resource constraints.

### 7.2 Limitations

Although this study has made certain explorations in theoretical perspectives and methodological techniques, subject to objective conditions, the following limitations remain that await further deepening in future research:

First, there are limitations regarding indicator representativeness and measurement granularity. It is difficult for any quantitative indicator to fully cover complex social realities. For instance, while using “effective urban resident wellbeing” as a proxy for social compliance possesses strong explanatory power in the Chinese context, it may not perfectly measure micro-level community mutual aid psychology. Furthermore, restricted by the availability of fine-grained city-level data during the crisis, digital governance capacity was operationalized as a binary condition (0/1) based on the presence of platforms. We acknowledge that this dichotomization inevitably masks substantial variations in system quality, coverage, and actual adoption rates among different cities, which future studies should address using multidimensional scales.

Second, the cross-sectional data features limit the observation of temporal dynamics. This study utilized the average values from 2020 to 2022 for analysis. While this temporal smoothing approach successfully captures macro-level configurational stability, it struggles to reflect within-city dynamic variations across different pandemic waves, policy phases (e.g., initial outbreak vs. zero-COVID vs. reopening), or viral mutations. Future research could employ longitudinal or Temporal QCA (T-QCA) to explore how these configurational pathways evolve over time.

Third, there are constraints regarding statistical inference at the methodological level. Although this study introduced bootstrap technology within the RStudio environment to enhance statistical robustness, it is essentially a resampling validation that cannot fundamentally break through the natural limits of small-N research. To further address concerns regarding the subjectivity of outcome variable construction, we conducted supplementary sensitivity analyses on the weighting scheme. By testing alternative plausible weights (e.g., equal weighting, detailed in Supplementary Table 4), we confirmed that the resulting configurational pathways remained highly consistent. This confirms that the governance patterns identified represent robust empirical patterns rather than artifacts of subjective weighting selections.

Finally, this study focuses on a sample of 23 Chinese cities, meaning the identified configurations possess specific boundary conditions. China's distinct governance context, characterized by top-down political mandates, a single-party state structure, specific crisis-response strategies (e.g., the zero-COVID policy), and robust party committees at the grassroots level, acts as a strong overarching confounder that significantly enhances organizational mobilization and social compliance during extreme crises. Therefore, the findings should be interpreted as context-specific insights rather than universal laws. The applicability and transferability of these pathways to countries with highly decentralized political systems or different socio-cultural environments remain to be tested through future cross-national comparative studies.

## Data Availability

Publicly available datasets were analyzed in this study. This data can be found here: The datasets analyzed for this study are publicly available across multiple official platforms and statistical yearbooks in China. Key macroscopic data sources include the National Bureau of Statistics of China (http://www.stats.gov.cn/english/), the Civil Aviation Administration of China (http://www.caac.gov.cn/en/SY/), and the official websites of respective municipal health commissions. Due to the multi-source nature of the urban governance data, a single compiled repository link or accession number is not applicable. Detailed references and sources for all condition and outcome variables are comprehensively listed in the manuscript's reference section.
