# Supplementary material for: What drives urban pandemic control performance in China? A configurational analysis of the resource-performance paradox
Source: Front Public Health. 2026 Jun 15;14:1849206. doi: 10.3389/fpubh.2026.1849206 (PMC13311025; doi:10.3389/fpubh.2026.1849206)
Supplement: Supplementary file 1 [file Table_1.docx]

# **Supplementary Table 1. Calibration Anchors and Rationales for Outcome Variables**

| **Sub-Dimension** | **Measurement Criteria**  **(Range)** | **Fuzzy Membership Score** | **Empirical Evidence** |
| --- | --- | --- | --- |
| 1. Impact Severity | 0–50 cases | 0.05–0.10 | **Wuxi:** Over 100 cumulative cases in its severest wave during the three-year period; assigned **0.15**.  **Wuhan:** During the most severe wave of the pandemic in 2020, over 50,000 cumulative cases were recorded; assigned **0.95**. |
|  | 51–200 cases | 0.11–0.20 |  |
|  | 201–500 cases | 0.21–0.35 |  |
|  | 501–999 cases | 0.36–0.49 |  |
|  | 1,000 cases | 0.50 (Crossover) |  |
|  | 1,001–2,500 cases | 0.51–0.65 |  |
|  | 2,501–9,999 cases | 0.66–0.80 |  |
|  | 10,000–49,999 cases | 0.81–0.94 |  |
|  | 50,000 cases | 0.95 |  |
|  | > 50,000 cases | 0.96–1.00 |  |
| 2. Lockdown Status | > 60 days | 0.96–1.00 | **Hangzhou:** No city-wide large-scale lockdown occurred in three years; assigned 0.05.  **Changchun:** City-wide lockdown lasted for 48 days in 2022; assigned 0.90. |
|  | 30–60 days | 0.80–0.94 |  |
|  | 14–30 days | 0.50–0.79 |  |
|  | 7–14 days (or large-area lockdown) | 0.10–0.49 |  |
|  | < 7 days (or no city-wide lockdown) | 0.05–0.09 |  |
| 3. Economic Disturbance | Avg. deviation is positive or near 0 | 0.05 | **Xiamen:** Average deviation was 2.03%; assigned 0.05.  **Wuhan:** Average deviation was -3.39%; assigned 0.90 (Note: Higher score indicates higher disturbance). |
|  | Avg. deviation between -0.5% and -2% | 0.50 (Crossover) |  |
|  | Avg. deviation below -2% | 0.95 |  |

# **Supplementary Table 2. Calibration Anchors and Rationales for Condition Variables**

| **Variable Code** | **Descriptive Statistics**  **(Raw Data)** | | **Full Non-Membership (0.05)** | **Crossover Point (0.50)** | | **Full Membership (0.95)** | **Rationale for Setting** |
| --- | --- | --- | --- | --- | --- | --- | --- |
| GDPpc | min: 5.36 | max: 18.41 | 8.0 | 12.5 | 16.5 | | Based on the national three-year average per capita GDP (CNY 79,600) and combined with the distribution of sample data. |
|  | mean: 11.84 | median: 11.73 |  |  |  |  |  |
|  | q1: 8.90 | q3: 14.64 |  |  |  |  |  |
| HealthExp  Change | min: 0.0067 | max: 0.059 | 0.007 | 0.012 | 0.04 | | Based on the national average annual change in health fiscal expenditure (0.0111) and combined with the distribution of sample data. |
|  | mean: 0.0187 | median: 0.0157 |  |  |  |  |  |
|  | q1: 0.0116 | q3: 0.0203 |  |  |  |  |  |
| Passenger Throughput | min: 0 | max: 3876.78 | 200 | 1,000 | 4,000 | | Adopting the CAAC’s classification thresholds for mainland Chinese airports (i.e., “10-million-level” and “2-million-level” passenger throughput), combined with sample data distribution. |
|  | mean: 1440.34 | median: 1216.91 |  |  |  |  |  |
|  | q1: 721.84 | q3: 1723.86 |  |  |  |  |  |
| Effective Urban Resident Well-being | Binary variable (0/1) | |  |  |  | | Assigned 1 if the city was listed in the “China’s Happiest Cities” ranking at least twice during 2020-2022; otherwise assigned 0. |
| DigitalGov | Binary variable (0/1) | |  |  |  | | Assigned 1 if the city’s government-led digital governance platform or health code system played a pivotal role beyond basic functions during the epidemic; otherwise assigned 0. |

***Note:*** *GDPpc unit: CNY 10,000; Passenger Throughput unit: 10,000 person-trips; q1:* *quartiles1;*

*a minimum passenger throughput volume of zero indicates that the city has no airport.*

# **Supplementary Table 3. Truth Table Analysis**

| **ID** | **GDPpc_fs** | **HealthExpChange**  **_fs** | **DigitalGov_cs** | **EURW**  **_cs** | **Passengerthroughput**  **_fs** | **OUT** | **N** | **Incl** | **PRI** | **Cases** |
| --- | --- | --- | --- | --- | --- | --- | --- | --- | --- | --- |
| 1 | 1 | 1 | 1 | 1 | 0 | 1 | 3 | 1.000 | 1.000 | Ningbo，Suzhou，Wuxi |
| 2 | 1 | 0 | 1 | 1 | 1 | 1 | 2 | 0.992 | 0.982 | Changsha，Nanjing |
| 3 | 0 | 0 | 1 | 0 | 0 | 1 | 1 | 0.951 | 0.879 | Hefei |
| 4 | 1 | 1 | 1 | 1 | 1 | 1 | 3 | 0.891 | 0.797 | Hangzhou，Qingdao，Guangzhou |
| 5 | 0 | 1 | 1 | 1 | 1 | 0 | 1 | 0.866 | 0.555 | Chengdu |
| 6 | 0 | 0 | 0 | 0 | 0 | 0 | 2 | 0.850 | 0.315 | Shijiazhuang，Lanzhou |
| 7 | 0 | 1 | 1 | 0 | 0 | 0 | 2 | 0.843 | 0.615 | Dongguan，Dalian |
| 8 | 0 | 0 | 1 | 0 | 1 | 0 | 1 | 0.753 | 0.261 | Zhengzhou |
| 9 | 0 | 1 | 0 | 0 | 0 | 0 | 3 | 0.729 | 0.267 | Changchun，Hohhot，Yinchuan |
| 10 | 1 | 1 | 1 | 0 | 1 | 0 | 3 | 0.685 | 0.449 | Xiamen，Shenzhen，Wuhan |
| 11 | 0 | 1 | 0 | 0 | 1 | 0 | 2 | 0.618 | 0.000 | Xi’an，Harbin |

# **Supplementary Table 4: Sensitivity analysis of fsQCA results under alternative outcome weighting schemes**

| **Parameter/Pathway** | **Original Weighting (45/35/20)** | **Equal Weighting (33/33/33)** |
| --- | --- | --- |
| Overall Consistency | 0.919 | 0.927 |
| Overall Coverage | 0.520 | 0.526 |
| Overall PRI Consistency | 0.869 | 0.870 |
| Identified Core Pathways | 3 configurations (grouped into 2 overarching patterns) | 3 configurations (Identical pathway structure) |
| Pattern 1:  Digital Empowerment | Supported (~GDPpc_fs*~HealthExpChange_fs*DigitalGov_cs*~UH_cs*~Passengerthroughput_fs) | Supported (Identical configuration identified) |
| Pattern 2:  Holistic Synergy | Supported  (Two sub-paths identified: GDPpc*HealthExpChange*DigitalGov*UH & GDPpc*DigitalGov*UH*Passengerthroughput) | Supported  (Identical sub-paths identified) |

# **Supplementary Table 5: A complete list of the 23 cities and their key characteristics**

| **City** | **Province** | **Economic Region** | **Administrative Status** | **GDPpc** |
| --- | --- | --- | --- | --- |
| Chengdu | Sichuan | Western | Provincial Capital | 9.22 |
| Xi’an | Shaanxi | Western | Provincial Capital | 8.41 |
| Yinchuan | Ningxia | Western | Provincial Capital | 10.10 |
| Hohhot | Inner Mongolia | Western | Provincial Capital | 8.90 |
| Lanzhou | Gansu | Western | Provincial Capital | 7.16 |
| Dalian | Liaoning | Northeast | Sub-provincial | 10.38 |
| Changchun | Jilin | Northeast | Provincial Capital | 7.50 |
| Harbin | Heilongjiang | Northeast | Provincial Capital | 5.36 |
| Ningbo | Zhejiang | Eastern | Sub-provincial | 14.99 |
| Hangzhou | Zhejiang | Eastern | Provincial Capital | 14.64 |
| Qingdao | Shandong | Eastern | Sub-provincial | 13.58 |
| Wuxi | Jiangsu | Eastern | Prefecture-level | 18.41 |
| Suzhou | Jiangsu | Eastern | Prefecture-level | 17.52 |
| Nanjing | Jiangsu | Eastern | Provincial Capital | 16.99 |
| Shijiazhuang | Hebei | Eastern | Provincial Capital | 5.80 |
| Shenzhen | Guangdong | Eastern | Sub-provincial | 17.27 |
| Guangzhou | Guangdong | Eastern | Provincial Capital | 14.59 |
| Dongguan | Guangdong | Eastern | Prefecture-level | 10.13 |
| Xiamen | Fujian | Eastern | Sub-provincial | 13.73 |
| Changsha | Hunan | Central | Provincial Capital | 12.74 |
| Wuhan | Hubei | Central | Provincial Capital | 13.45 |
| Zhengzhou | Henan | Central | Provincial Capital | 9.77 |
| Hefei | Anhui | Central | Provincial Capital | 11.73 |

***Notes:***

*The division of economic regions is based on China’s national economic policies, dividing the country into four major economic regions: Eastern, Central, Western, and Northeast China.*

*GDPpc represents the three-year average GDP per capita (2020–2022) based on the permanent resident population, expressed in units of 10,000 CNY.*
